# Assessment of sublethal and transgenerational effects of spirotetramat, on population growth of cabbage aphid, *Brevicoryne brassicae* L. (Hemiptera: Aphididae)

**DOI:** 10.3389/fphys.2022.1014190

**Published:** 2022-12-08

**Authors:** Ayesha Iftikhar, Faisal Hafeez, Muhammad Asif Aziz, Muhammad Hashim, Afifa Naeem, Hafiz Kamran Yousaf, Muhammad Jawad Saleem, Sabir Hussain, Muhammad Hafeez, Qurban Ali, Muzammal Rehman, Sumreen Akhtar, Romina Alina Marc, Khalid M. Al Syaad, Yasser Sabry Mostafa, Fatimah A. Al Saeed

**Affiliations:** ^1^ Entomological Research Institute, Ayub Agricultural Research Institute, Faisalabad, Pakistan; ^2^ Department of Entomology, Faculty of Crop and Food Sciences, Pir Mehr Ali Shah, Arid Agriculture University, Rawalpindi, Pakistan; ^3^ Department of Entomology, Faculty of Agricultural Sciences, University of the Punjab, Lahore, Pakistan; ^4^ Department of Zoology, University of Okara, Okara, Pakistan; ^5^ Department of Agriculture, Mir Chakar Khan Rind University, Sibi, Pakistan; ^6^ State Key Laboratory of Rice Biology and Ministry of Agriculture Key Lab of Molecular Biology of Crop Pathogens and Insects, Institute of Insect Sciences, Zhejiang University, Hangzhou, China; ^7^ Key Laboratory of Plant Genetics and Breeding, College of Agriculture, Guangxi University, Nanning, China; ^8^ Department of Zoology, Faculty of Basic Sciences, University of the Punjab, Lahore, Pakistan; ^9^ Food Engineering Department, Faculty of Food Science and Technology, University of Agricultural Science and Veterinary Medicine Cluj-Napoca, Cluj-Napoca, Romania; ^10^ Biology Department, Faculty of Science, King Khalid University, Abha, Saudi Arabia; ^11^ Department of Biology, College of Science, King Khalid University, Abha, Saudi Arabia; ^12^ Department of Biology, Saudi Arabia Research Center for Advanced Materials Science (RCAMS), College of Science, King Khalid University, Abha, Saudi Arabia

**Keywords:** cabbage aphid, population growth, spirotetramat, sublethal concentrations, transgenerational effects

## Abstract

The cabbage aphid (*Brevicoryne brassicae* L.) is a devastating pest of cruciferous crops causing economic damage worldwide and notably owing to its increasing resistance to commonly used pesticides. Such resistance prompts the development of integrated pest management (IPM) programs that include novel pesticides being effective against the aphids. Spirotetramat is a novel insecticide used against sap-sucking insect pests, particularly aphids. This study evaluated the toxicity of spirotetramat to adult apterous *B. brassicae* after 72 h using the leaf dipping method. According to the toxicity bioassay results, the LC_50_ value of spirotetramat to *B. brassicae* was 1.304 mgL^−1^. However, the sublethal concentrations (LC_5_ and LC_15_) and transgenerational effects of this novel insecticide on population growth parameters were estimated using the age-stage, two-sex life table theory method. The sublethal concentrations (LC_5_; 0.125 mgL^−1^ and LC_15_; 0.298 mgL^−1^) of spirotetramat reduced the adult longevity and fecundity of the parent generation (F_0_). These concentrations prolonged the preadult developmental duration while decreasing preadult survival, adult longevity and reproduction of the F_1_ generation. The adult pre-reproductive period was also extended by spirotetramat treatment groups. Subsequently, the population growth parameters such as the intrinsic rate of increase *r*, finite rate of increase *λ* and net reproductive rate *R*_0_ of the F_1_ generation were decreased in spirotetramat treatment groups whereas, the mean generation time *T* of the F_1_ generation was not affected when compared to the control. These results indicated the negative effect of sublethal concentrations of spirotetramat on the performance of *B. brassicae* by reducing its nymphal survival, extending the duration of some immature stages and suppressing the population growth of *B. brassicae*. Overall, we demonstrated that spirotetramat is a pesticide showing both sublethal activities, and transgenerational effects on cabbage aphid; it may be useful for implementation in IPM programs against this aphid pest.

## Introduction

The cabbage aphid, *Brevicoryne brassicae* Linnaeus (Hemiptera: Aphididae), is one of the most destructive pests of the Brassicaceae family and can be found around the globe (Anzabi et al., 2014; Sarhozaki and Safavi, 2014; Ahmed et al., 2020; Shonga and Getu, 2021a; Shonga and Getu, 2021b) including Pakistan (Abbas et al., 2017). It causes damage directly by sucking the plants almost in all growth stages and indirectly by transmitting diseases or secreting honeydew (Bashir and Azim, 2013). It is also known to be the vector of various plant viruses (Dáder et al., 2017). Although various management technologies have been developed and implemented against *B. brassicae* (e.g., promoting biocontrol services, (Lu et al., 2012), its management still primarily relies on the application of pesticides (Shang et al., 2012; Roh et al., 2015).

Many new insecticides have been developed and commercially available that are safer for the environment and human health and control insect pests more effectively (Babcock et al., 2011). Spirotetramat, a tetramic acid-based insecticide with a novel mode of action, belonging to a new cyclic keto-enol compound developed by Bayer Crop Science is being used worldwide against aphids, mites and other piercing-sucking pests of crops (Brück et al., 2009; Wang et al., 2016). It has distinctive translocation properties in that after foliar application; it is simultaneously translocated upwards by the xylem and downwards through the phloem (Brück et al., 2009). Spirotetramat acts as a lipid biosynthesis inhibitor that reduces the fecundity and fertility of sucking insect pests (Gong et al., 2016a; Salazar-López et al., 2016). The lipids are of vibrant significance to many insects for metamorphosis, embryogenesis and flight (Arrese et al., 2001). Due to the absence of cross-resistance to prevailing classes of chemical insecticides, spirotetramat may be a significant tool to achieve insecticide resistance in many crop pests around the globe (Ouyang et al., 2012; Döker et al., 2021). This has prompted the development of new pest management strategies and products, for example exploring and developing novel pesticides.

One of the main challenges in toxicology is how to evaluate the overall effect of toxic substances on insect populations (Lashkari et al., 2007). Traditional approaches to determining the lethal concentration of insecticides on insects are centered on assessing individual mortality in the short term; however, the elucidation of acquired data at the population level is inadequate due to the inadequacy of the number of endpoints (Stark and Banks, 2003; Desneux et al., 2007). After the application of insecticides in agricultural systems, insecticides may degrade to sublethal and low lethal doses over time in the field due to which some target pests do not show rapid mortality to the lethal dose (Mahmoodi et al., 2020; Ullah et al., 2020; Jie et al., 2021). Sublethal effects have been described as effects on the physiology and behavior of an individual that survives exposure to an insecticide or toxin at the sublethal or lethal dose/concentration (Desneux et al., 2007). Numerous studies have been carried out on this problem which showed that insect pests exposed to these lethal or sublethal doses or concentrations of toxicants go through several physiological and behavior impairments, hormesis, and better tolerance for chemical materials (Desneux et al., 2006; Tan et al., 2012; Guedes et al., 2016; Yousaf et al., 2018; Lv et al., 2021; Zhang et al., 2021). Besides mortality, sublethal effects of insecticides may be manifested in many ways, such as biological and behavioral parameters including developmental time, fecundity, longevity, sex ratio, feeding activity, predation rate, orientation, and mobility. (Desneux et al., 2007; Guedes et al., 2016). and may result not only from direct contact with herbicides but also as a result of feeding on contaminated food. Moreover, positive and negative effects triggered by the sublethal doses of insecticides can be transmitted from offspring to several filial generations (Hercus and Hoffmann, 2000; Shikano, 2017; Mahmoodi et al., 2020).

It is necessary to assess the sublethal effects to optimize the application of insecticides along with their toxic effects to evaluate their effectiveness precisely (Stark and Banks, 2003). Attaining this evidence can help in explaining the cases of insect outbreaks and pest reappearance as a result of prior pesticide applications. Another advantage of the complete assessment of the sublethal effects of pesticides would be getting the capability to develop more effective and environment-friendly procedures (Desneux et al., 2007; Rahmani and Bandani, 2013; Sohrabi et al., 2013). Demographic toxicology is an ecotoxicological approach that assimilates the life history and life table in the circumstantial of toxicology (Sarhozaki and Safavi, 2014). It helps appraise the sublethal special effects of a pesticide on the anticipated population development of the embattled pests (Stark and Banks, 2003; Lashkari et al., 2007). Insect life tables are helpful and dynamic tools for the estimation of sublethal effects of synthetic and natural insecticides on insect pests (Cutler et al., 2009; Saska et al., 2016; Liang et al., 2019). This is because their limitations openly imitate the wide-ranging effect of biological features (i.e., survival, reproduction, development, and sex ratio) on population fitness. Sublethal effects of many classes of insecticides, i.e., pymetrozine, imidacloprid, thiamethoxam, thiamethoxam-lambda cyhalothrin, buprofezin, and acetamiprid (Lashkari et al., 2007; Sarhozaki and Safavi, 2014) and (Mahmoodi et al., 2020) has been studied on cabbage aphid.

To date, no reports have been found regarding sublethal and transgenerational effects of spirotetramat on *B. brassicae*. In the present study we investigated within- and transgenerational (maternal) effects of sublethal insecticide stress on several fitness-associated traits; survival, and development time to understand the relations between the exposure doses of spirotetramat and insect response at both the individual and population levels.

## Materials and methods

### Insect rearing

The cabbage aphid was used as a study insect and was collected from brassica crop (*Brassica napus* L. var. canola) grown at the research area of Ayub Agricultural Research Institute, Faisalabad (31.4041° N, 730,487° E). The stock culture of *B. brassicae* was established on insecticide-free leaves of brassica plants under standard circumstances (24 ± 1°C temperature, 70 ± 5% relative humidity and 16:8 h light-dark period) at Entomological Research Institute, Faisalabad. Insecticide-free brassica plants were replaced every week.

### Chemical and toxicity bioassays

The spirotetramat (CAS No. 203313-25-1; Movento^®^ 240 SC; 240 g/L active ingredients) was obtained from Bayer Crop Science Co. Ltd. (Australia). The insecticide tested in this study is registered and being used in brassica crops in Pakistan to manage aphids. Toxicity bioassays were conducted with apterous adult aphids using the leaf dip method described by (Ullah et al., 2020) to measure the lethal and sublethal toxicity of spirotetramat. The distilled water was utilized to make spirotetramat concentrations (6, 3, 1.5, 0.75 and 0.375 mgL−1). Distilled water dipped leaf discs were used only for the control group. Brassica leaf discs were cut by a sharp metal cylinder and dipped in the dilutions of respective insecticide solution for 30 s. The treated leaf discs were placed at room temperature for 30 min to dry residual solution droplets on the leaves. The treated leaves were placed in plastic Petri dishes (size: 3 × 1.5 cm, with a biaxial surface downward) lined with moistened filter paper. Each concentration comprised three replications of 30 apterous adult aphids (≤24 h old) and five leaves were used for each replication. Mortality data were estimated at 72 h of spirotetramat exposure. Aphids were scored as dead if they did not exhibit repetitive (i.e., non-reflex) movement when gently probed with a soft camel hair brush (Moores et al., 1996). The lethal concentrations LC_5_, LC_15,_ and LC_50_ were calculated by using PoloPlus 2.0 software (LeOra Software Inc. Berkely, CA).

### Sublethal response of spirotetramat on F_0_ generation

Approximately 650 adult aphids were transferred to insecticide-free brassica leaves. All adult apterous aphids were removed after 72 h, while the neonate nymphs were retained on the leaves for 08 days to become adults. This procedure was applied to ensure the same age group of aphids before exposure to the insecticide. Therefore, the LC_5_ (0.125 mgL^−1^), as well as LC_15_ (0.298 mgL^−1^) concentrations of spirotetramat, were used in this study to evaluate their impact on the F_0_ generation of *B. brassicae* and distilled water was used as control. Brassica leaf discs were uniformly dipped in LC_5_ and LC_15_ of spirotetramat and control solution for 30 s, air-dried for 30 min. Then the dry treated leaf discs were placed in plastic Petri dishes with their biaxial surface downward containing moistened filter paper to maintain humidity. Adult apterous aphids were released to feed on these treated leaves and control solution for 72 h. After that, sixty healthy and surviving aphids were transferred to untreated fresh leaf discs in plastic Petri dishes individually. Adults of the F_0_ generation were inspected daily for recording longevity and fecundity, while the newborn nymphs were removed until the adult died. The leaf discs were changed every 3–4 days to prevent fungal growth until the adult aphids died.

### Sublethal response of spirotetramat on the F_1_ generation

New-born nymphs (age ˂24 h) obtained from F_0_ adults, were gathered as F_1_ generation and then transferred to Petri-dishes independently. These aphids (F_1_ generation) were individually reared on insecticide-free brassica leaf discs, as described in the previous F_0_ generation. This method was repeated 60 times for the spirotetramat treatments (LC_5_ and LC_15_) and the control, treating each aphid as a single replication. Survival, development and reproduction were noted on daily basis. During the reproductive period, newborn nymphs were counted-up and then removed. Fresh leaf discs were changed every 3–4 days until the death of the adult aphid.

### Statistical analysis

The bioassay data were used to calculate the lethal (LC_50_) and sublethal (LC_5_ and LC_15_) concentrations of spirotetramat by using Probit analysis (Finney, 1971) in PoloPlus 2.0 (Software, 2005). The life-history data of cabbage aphids exposed to sublethal concentrations of spirotetramat and the control were subjected to the computer-based program software (TWO SEX-MSChart) (Chi H, 2022) and analyzed by employing the age-stage two-sex life table theory (Chi and Liu, 1985; Chi, 1988). The life table parameters *s*_*xj*_, *l*_*x*_, *m*_*x*_, *e*_*xj*_ and *v*_*xj*_ (age-stage survival rate, age-specific survival rate, age-specific fecundity, age-stage life expectancy and age-stage reproductive value, respectively) were estimated (where *x* is the age and *j* is the stage of insect). The population parameters including *r*, *λ*, *R*_*0*_ and *T* (intrinsic rate of increase, finite rate of increase, net reproductive rate and mean generation time, respectively) were also estimated. The life expectancy (*e*_*xj*_) was determined according to (Chi and Su, 2006). The reproductive value (*v*_*xj*_) was calculated according to (Tuan et al., 2014a; 2014b). The standard bootstrap method was used with 100,000 resampling to calculate the variance as well as standard errors for biological and population growth parameters (Efron and Tibshirani, 1993; Akköprü et al., 2015). A paired-bootstrap-test at a 5% significance level based on the confidence interval of differences was used to analyze differences among treatments. The bootstrap along with the paired bootstrap test was also included in the TWO SEX-MSChart computer program.

## Results

### Toxicity of spirotetramat to apterous adult *B. brassicae*

The toxicity of spirotetramat against apterous adult cabbage aphid was determined after exposure for 72 h (Table 1). The estimated value of LC_50_ with a 95% confidence interval was 1.304 mgL^−1^, while LC_5_ and LC_15_ values were 0.125 mgL^−1^ and 0.298 mgL^−1^, respectively. The LC_5_ and LC_15_ values of spirotetramat obtained were selected to further estimate the sublethal as well as transgenerational effects of spirotetramat on demographic parameters of *B. brassicae*.

**TABLE 1:** Bioassay of Spirotetramat on apterous adults of *Brevicoryne brassicae*.

Insecticide	N^a^	Slope±S.E^b^	LC_5_ ^c^	LC_15_ ^c^	LC_50_ ^c^	χ^2^ (df)^d^	*p*	Regression equation
Spirotetramat	540	1.618 ± 0.162	0.125 (0.035–0.244)	0.298 (0.124–0.480)	1.304 (0.929–1.787)	3.32 (3)	0.344	y = 4.773 + 1.660x

^a^ N = number of apterous adult aphids exposed.

^b^ S.E. , standard error.

^c^ Expressed in mg L^−1^; 95% CI, of LC, are given in bracket.

^d^ Chi square and degree of freedom.

### Sublethal response of spirotetramat on longevity and fecundity of parent F_0_ generation of *B. brassicae*

The longevity and fecundity of test individuals (F_0_ generation) were affected when exposed for 72 h to the two sublethal concentrations of spirotetramat (LC_5_ and LC_15_) as compared with the control (Table 2). The longevity of *B. brassicae* adults was significantly reduced when treated with Spirotetramat at LC_15_ as compared to the control (*p* < 0.00001), while that recorded on adults treated with insecticide at LC_5_ did not show a significant difference. A similar trend of the longevity of *B. brassicae* adults was found between LC_5_ and LC_15_ (*p* < 0.00053). Furthermore, F_0_ adults showed significantly reduced fecundity in both treatments (LC_5_ and LC_15)_ as compared to the control (*p* < 0.00001). It was 31.75 nymphs/female in the control group, 17.31 nymph/female and 14.68 nymphs/female in the adults treated at LC_5_ and LC_15_ concentrations of the spirotetramat insecticide, respectively. There was a non-significant difference in fecundity between LC_5_ and LC_15_ treated groups.

**TABLE 2:** Effect of exposure of parent adults (F_0_ generation) of *Brevicoryne brassicae* to Spirotetramat at LC_5_ and LC_15_ on their longevity and fecundity (Mean ± SE).

Parameters	Control	LC_5_	LC_15_
Adult longevity (d)	10.10 ± 0.35 a	9.01 ± 0.41 ab	8.03 ± 0.40 b
Fecundity (nymphs/female)	31.75 ± 1.48 a	17.31 ± 0.85 b	14.68 ± 0.85 b

Different letters within the same row represent significant differences at *p* ˂ 0.05 (one way ANOVA, followed by Tukey’s HSD, test).

### Transgenerational sublethal effects of spirotetramat on biological parameters of the F_1_ generation of *B. brassicae*

Table 3 indicates the developmental duration, longevity and fecundity of the subsequent progeny generation (F_1_) of *B. brassicae* exposed to sublethal concentrations (LC_5_ and LC_15_) of spirotetramat. However, the duration of immature developmental stages, pre-adult duration, pre-adult survival, adult pre-reproductive period (APRP) and total pre-reproductive period (TPRP) of the F_1_ generation did not show significant differences among treated groups. The values of adult longevity, total longevity and fecundity were recorded higher in control treatment as compared to the populations treated with sublethal concentrations of spirotetramat. Significantly lower values of adult longevity and total longevity between LC_5_ and LC_15_ as compared to the control (*p* < 0.00001), similarly significantly lower fecundity were observed in populations treated with spirotetramat at LC_5_ and LC_15_ as compared to the control (*p* < 0.00001).

**TABLE 3:** Developmental duration, longevity and fecundity of different stages for F_1_ generation *B. brassicae* after exposure of parental adult (F_0_) to the LC_5_ and LC_15_ of Spirotetramat.

Treatments						
Stages (d)	N	Control	N	LC_5_	N	LC_15_
		Mean ± SE		Mean ± SE		Mean ± SE
1st Instar (N_1_)	60	1.44 ± 0.07a	60	1.57 ± 0.07a	60	1.57 ± 0.07a
2nd Instar (N_2_)	55	1.68 ± 0.08a	51	1.61 ± 0.09a	49	1.70 ± 0.1a
3rd Instar (N_3_)	53	2.37 ± 0.09a	49	2.43 ± 0.1a	46	2.41 ± 0.12a
4th Instar (N_4_)	51	2.49 ± 0.09a	47	2.54 ± 0.11a	44	2.59 ± 0.1a
Pre-adult	51	7.96 ± 0.18a	46	8.24 ± 0.18a	44	8.25 ± 0.2a
Pre-adult survival		0.85 ± 0.04a		0.76 ± 0.05a		0.73 ± 0.05a
Adult longevity	51	12.45 ± 0.24a	46	10.11 ± 0.21b	44	9.05 ± 0.22c
Total longevity	51	20.41 ± 0.31a	46	18.35 ± 0.28b	44	17.3 ± 0.22c
APRP		0.88 ± 0.1a		0.93 ± 0.1a		0.89 ± 0.11a
TPRP		8.84 ± 0.2a		9.17 ± 0.22a		9.14 ± 0.21a
Reproductive days		10.51 ± 0.19a		8.11 ± 0.17b		7.36 ± 0.15c
Fecundity (nymphs/female)		35.49 ± 0.58a		27.41 ± 0.55b		23.02 ± 0.62c

Means within the same row followed by same lowercase letters represent that treatments are not significantly different (*p* ˂ 0.05) from each other based on paired bootstrap test. Standard errors (SE) were estimated by 100,000 resampling using the bootstrap technique in TWOSEX-MSChart.

### Transgenerational sublethal effects of spirotetramat on population growth parameters of the F_1_ generation of *B. brassicae*

**FIGURE 1:**
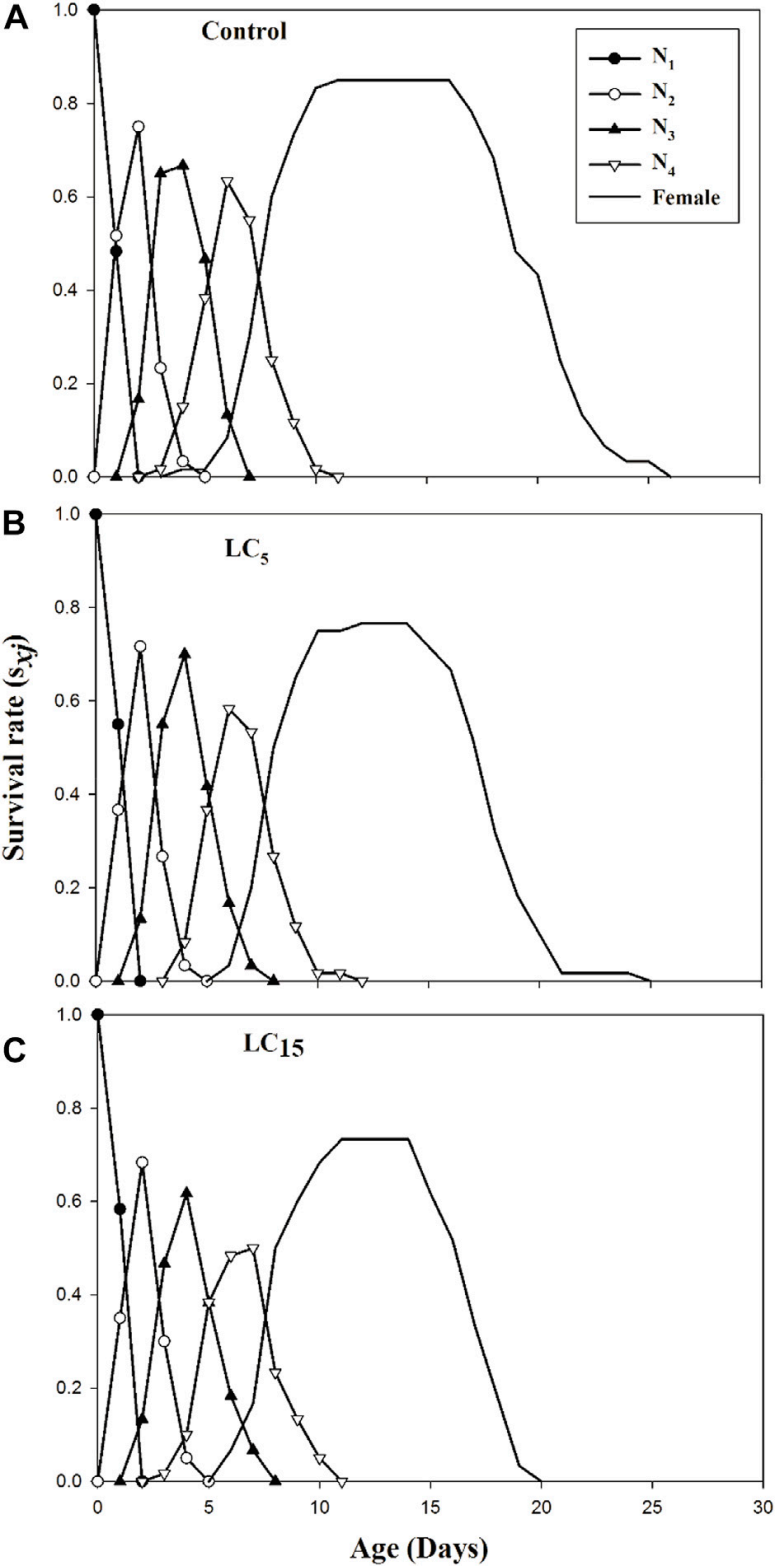
**(A–C)**: Age-stage specific survival rate (*s*_*xj*_) of control group **(A)** and *B. brassicae* treated with LC_5_ **(B)** and LC_15_ **(C)** of Spirotetramat in F_1_ generation.

**FIGURE 2:**
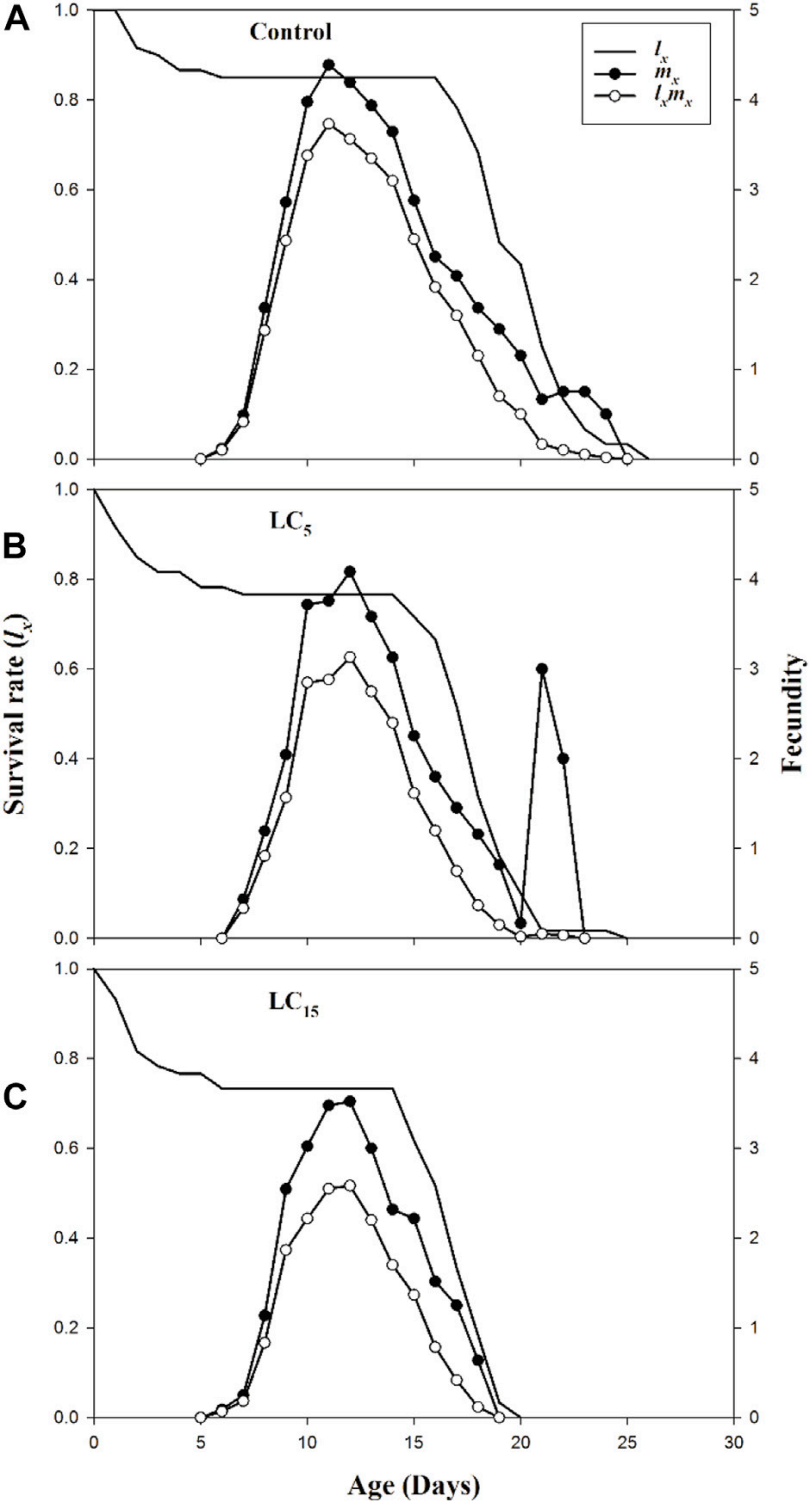
**(A–C)**: Age specific survival rate (*l*_*x*_), age specific fecundity (*m*_*x*_) and net maternity (*l*_*x*_ *m*_*x*_) of control group **(A)** and *B. brassicae* treated with LC_5_ **(B)** and LC_15_ **(C)** of Spirotetramat in F_1_ generation.

The stage differentiation and overlapping survival curves in the F_1_ generation of *B. brassicae* exposed to LC_5_ and LC_15_ of spirotetramat are shown in Figure 1(A–C). Age-stage specific survival rate *s*_*xj*_ is the expected duration of neonate nymphs that will survive to age *x* and stage *j*. The probability of reaching the adult stage was 0.73, 0.75, and 0.83 for a neonate nymph from LC_15_, LC_5_ and control groups, respectively. Age-specific survival rate (*l*_*x*_), age-specific fecundity (*m*_*x*_) and age-specific net maternity (*l*_*x*_ *m*_*x*_) for LC_5_, LC_15_ and control treatments of *B. brassicae* are presented in Figure 2(A–C). The age-specific survival rate (*l*_*x*_) of *B. brassicae* decreased with age *x* and the maximum survival period for LC_5_ and LC_15_ concentrations of spirotetramat were 15 days and 14 days, respectively. This was lower than the maximum survival period of the control group (16 days). The age-specific fecundity (*m*_*x*_) curves for LC_5_ (4.08 offspring and 3.00 offspring) at age of 12 and 21 days while *m*_*x*_ curve for LC_15_ (3.52 offspring) occurred at the age of 12 days, compared with the control (4.39 offspring) at age of 11 days. The maximum values of age-specific maternity (*l*_*x*_ *m*_*x*_) for LC_5_, LC_15_ and control were 3.73, 3.13, and 2.58 offspring, respectively.

**FIGURE 3:**
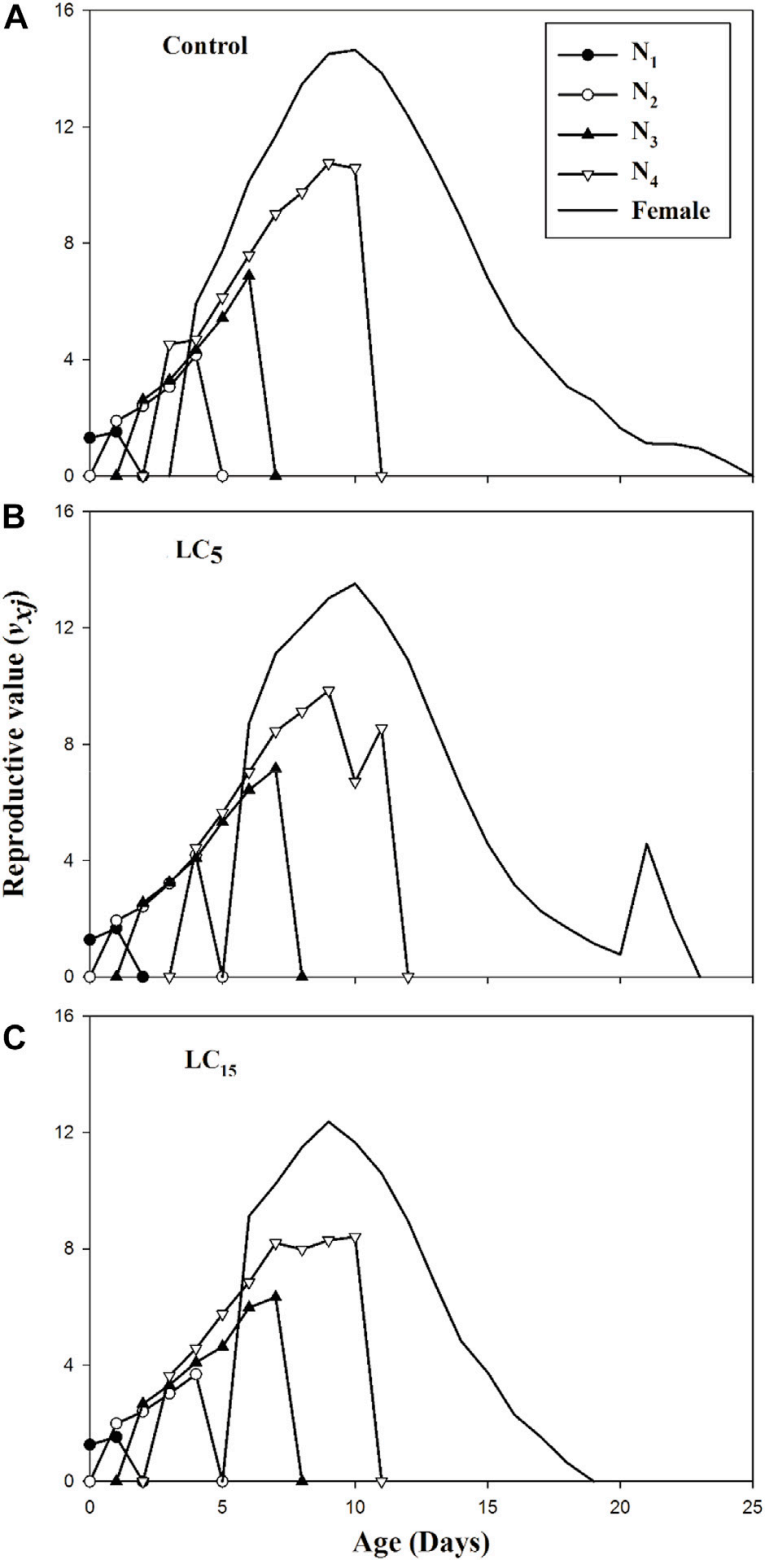
**(A–C)**: Age-Stage specific reproductive value (v_xj_) of control group **(A)** and B. brassicae treated with LC_5_ **(B)** and LC_15_ **(C)** of Spirotetramat in F_1_ generation.

The age-stage specific life expectancy (*e*_*xj*_) represents the expected lifespan of individual *B. brassicae* exposed to spirotetramat treated populations (LC_5_ and LC_15_) and control (Figures 3A–C).

**FIGURE 4:**
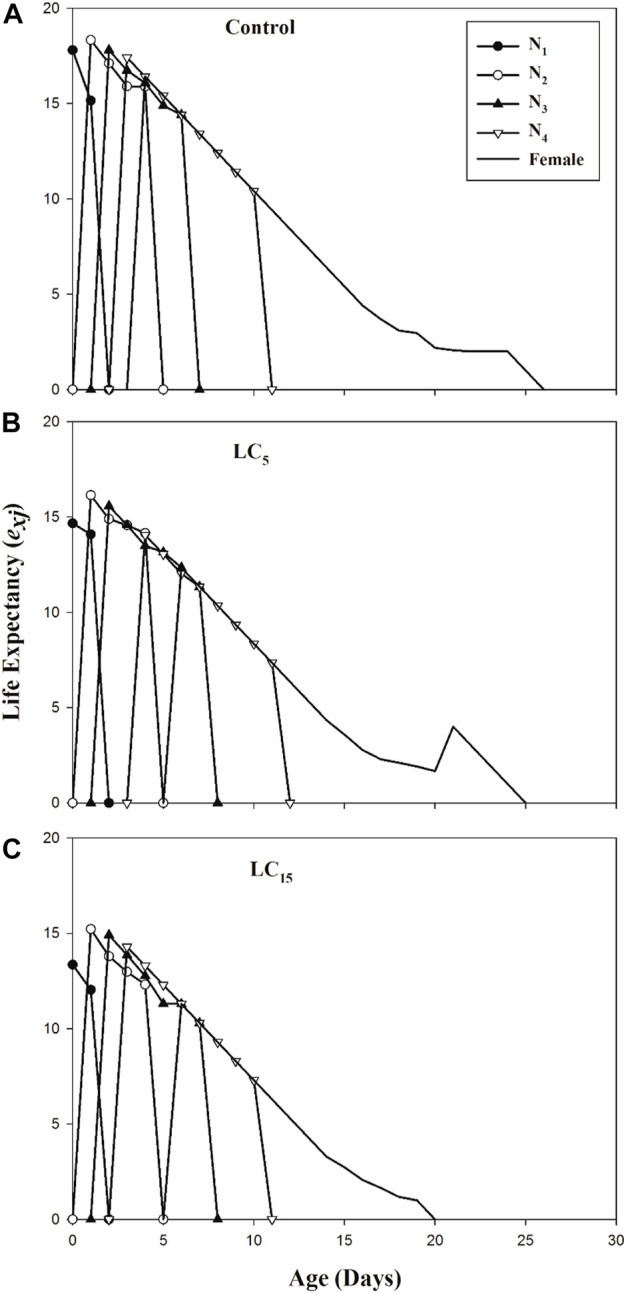
**(A–C)**: Age-stage specific life expectancy (e_xj_) of control group **(A)** and B. brassicae treated with LC_5_ **(B)** and LC_15_ **(C)** of Spirotetramat in F_1_ generation.

The *e*_*xj*_ curves showed that individuals in the control group of F_1_ generation are expected to survive longer than the spirotetramat treated population (LC_5_ and LC_15_). The age-stage specific reproductive value (*v*_*xj*_) exhibits the prediction of future offspring for individuals of *B. brassicae* from age *x* to stage *j* (Figures 4A–C). The highest reproductive value (*v*_*xj*_) peak was identified in the control group (*v*_*10*_ = 14.64), the peak value obtained for LC_5_ treated population was different from the control group although occurred on the same day (*v*_*10*_ = 13.52) while the earliest peak was observed in LC_15_ treated population (*v*_*9*_ = 12.36).

Population growth parameters of the F_1_ generation of *B. brassicae* treated with sublethal concentrations of the insecticide are shown in Table 4. The mean generation time (*T*) did not affect all the treatments. Moreover, the net reproductive rate (*R*_0_) was reduced from 30.16 offspring/individual in the control group to 16.88 offspring/individual in the population treated with LC_5_ and LC_15_ of the insecticide (*p* < 0.00001). The intrinsic rate of increase (*r*) (*p* < 0.00743) and finite rate of increase (*λ*) (0.00727) of F_1_ individuals were reduced at both sublethal concentrations of insecticide (LC_5_ and LC_15_) in comparison to those of the control group.

**TABLE 4:** Sublethal effects of Spirotetramat on population growth parameters (Mean ± SE) of F_1_ generation of *B. brassicae*.

Treatments			
Population parameters	Control	LC_5_	LC_15_
*T* (d)	12.67 ± 0.21a	12.55 ± 0.21a	12.27 ± 0.63a
*R*_0_ (offspring/individual)	30.16 ± 1.70a	21.01 ± 155b	16.88 ± 1.38c
*r* (d^−1^)	0.2688 ± 0.0064a	0.2426 ± 0.0073b	0.2302 ± 0.0080b
*λ* (d^−1^)	1.3084 ± 0.0084a	1.2746 ± 0.0093b	1.2588 ± 0.0101b

Where; *T* = mean generation time, *R*_0_ = net reproductive rate, *r* = intrinsic rate of increase, *λ* = Finite rate of increase. Means within the same row followed by same lowercase letters represent that treatments are not significantly different (*p* ˂ 0.05) from each other based on paired bootstrap test. Standard errors (SE) were estimated by 100,000 resampling using the bootstrap technique in TWOSEX-MSChart.

## Discussion

Resistance of *B. brassicae* has been reported to many organophosphates and pyrethroids (Ahmad and Akhtar, 2013). Due to this farmers have to increase the frequency of insecticide application which intern causes more resistance to these insecticides and also increases environmental pollution (Liang et al., 2019). Thus the study of this innovative substitute, Spirotetramat, is crucial in adjourning the increase of resistance to *B. brassicae*. The information regarding sublethal responses of this novel chemical has been reported on various insects, including *Myzus persicae* (Wang et al., 2016), *Aphis gossypii* (Gong et al., 2016b), *Tetranychus urticae* (Marcic et al., 2012), *Encarsia Formosa* (Drobnjaković and Marčić, 2021) and *Cryptolaemus montrozuieri* (Planes et al., 2013) but the information regarding resistance to a novel mode of insecticides action is lacking (Ahmed et al., 2020). In the present study, potential sublethal and transgenerational effects of spirotetramat on *B. brassicae* were assessed for two succeeding generations (F_0_ and F_1_). In a previous study, spirotetramat acts gentler, however, with the main influence on undeveloped phases and expressively disturbs the fertility and fecundity of *M. persicae* by triggering a high fraction of nonviable nymphs (Wang et al., 2016).

In the current study, all life table parameters of the filial generation, *r*, *λ*, and *R*_0_, as well as development, fecundity, duration of oviposition period, longevity and survival of both the treated and filial generations, were negatively and progressively affected by the LC_5_ and LC_15_ concentration of the spirotetramat insecticide. Similar adverse effects were also reported when sublethal concentrations of flupyradifurone were exposed in the F_0_ generation of *M. persicae* (Tang et al., 2019) and F_1_ generation of cotton aphid, *A. gossypii* (Liang et al., 2019). (Qu et al., 2015) reported that the fecundity and longevity of apterous aphid *A. gossypii* was expressively lowered after exposure to sublethal concentration of imidacloprid. Furthermore, the progeny of apterous female adults of *A. gossypii* was reduced when exposed to cycloxaprid and nitenpyram (Wang et al., 2017; Yuan et al., 2017; Cui et al., 2018). Sulfoxaflor also reduced the fecundity in the F_0_ generation of *Sogatella frucifera* (Xiang et al., 2019) and *Nilaparvata lugens* (Liao et al., 2019). Moreover, negative effects such as fecundity of *Apolygus lucorum* and longevity of *Bemisia tabaci* were decreased drastically when exposed to low or sublethal concentrations of cycloxaprid and buprofezin (Sohrabi et al., 2011; Pan et al., 2014). All information thoroughly described the negative impact of insecticide concentrations (low or sublethal), which most probably occurred in the field after the degradation of insecticide over time (Desneux et al., 2005; Desneux et al., 2007; Hafeez et al., 2021b). This mechanism might be linked with the vigor coordination in insects after exposure to insecticides and additional drive has been subjugated by insects to manage the insecticide compression, resulting in a shortage of energy for productivity. Also, the diminution trend in fecundity and longevity specified a dearth of hormetic effects, which develops an imperative sublethal outcome of insecticides (Liang et al., 2019). Hormesis can be explained as a dose-response association categorizing over the reverse of the reaction between low and high-stress doses (Jager et al., 2013; Guedes and Cutler, 2014). In the previous study, hormesis has been observed in various insect species and insecticides, likely the higher fecundity in *M. persicae* exposed to imidacloprid (Ayyanath et al., 2013) and the outbursts in *Oligonchus ilicis* tempted by pyrethroid (Cordeiro et al., 2013). Sublethal and transgenerational effects of spirotetramat affected developmental physiology, indicating the role of sublethal concentrations in larval growth, development, and sensitivity to spirotetramat. In previous studies, Physiological and biochemical studies have shown that P450 enzymes are vital to in insect hormone metabolism pathways but details of the molecular processes remain unknown (Iga and Kataoka, 2012; Hafeez et al., 2022). The growth and developmental physiology of *B. brassicae* was hindered after being treated with sublethal concentrations of spirotetramat, but how these sublethal concentrations regulate this process requires further study.

In the current study, the evaluation of transgenerational effects in the filial F_1_ cohort of *B. brassicae*, imitated that the exposure to LC_5_ and LC_15_ of spirotetramat in the parent generation (F_0_) inclined the F_1_ generation population growth, particularly through an amplified preadult developmental length and total pre-reproductive period (TPRP). Comparable outcomes originated in *A. gossypii* when treated with methyl benzoate, thiamethoxam, and flonicamid (Mostafiz et al., 2020; Ullah et al., 2020; Shi et al., 2022). In previous studies, sublethal effects of thiamethoxam insecticides have also been reported on the population development of *Bradysia odorriphaga* (Zhang et al., 2014) and *Hippodamia variegate* (Rahmani and Bandani, 2013). A similar trend of sublethal effects of imidacloprid was described on *B. tabaci* (He et al., 2013a), *A. lucorum* (Tan et al., 2012), *M. persicae* and *B. brassicae* (Lashkari et al., 2007; Wang et al., 2008). Some neonicotinoid insecticides (such as nitenpyram, clothianidin, acetamiprid and thiacloprid) can lead to substantial adverse effects on the biological traits of *A. gossypii* (Shi et al., 2011). (Lashkari et al., 2007) stated that treatment with imidacloprid lowered the average cohort time in *B. brassicae*. In *B. tabaci*, a low dose of imidacloprid did not disturb the biological and population growth factors but extended the mean generation time (Sohrabi et al., 2011). All these conclusions predicted that the effects of insecticide can vary intensely reliant on several aspects e.g., the amount of insecticide used, insecticide class, the insect species, the definite application circumstances, and the physiological state of the targeted organism (Shi et al., 2011; Han et al., 2012; Zhang et al., 2012; Guo et al., 2013; Xiao et al., 2015; Haddi et al., 2016).

The other parameters including *s*_*xj*_, *l*_*x*_ and *v*_*xj*_ were to clarify the conflicting effects of insecticides on population growth and the development of various insect pests (He et al., 2013b; Iftikhar et al., 2020; Hafeez et al., 2021a)*.* Because *l*_*x*_ is an elementary form of *s*_*xj*_, the *l*_*x*_ curve of the insecticide-exposed cluster could only exemplify that spirotetramat reserved the survival rate in the immature stages (Liang et al., 2019). (Chen et al., 2016) reported that a higher survival rate after age 33 days in the flupyradifurone-treated aphids exhibited a thinkable concealed hormesis. In our study, we demonstrated that hormesis is not a key factor in terms of effects of spirotetramat on *B. brassicae*. In addition to this, the decrease in *m*_*x*_ and *l*_*x*_ *m*_*x*_ curves of insecticide-treated groups reflected that the productiveness of the filial F1 generation is affected by insecticide (Tang et al., 2015; Liang et al., 2019). However, considering all biological processes at play when arthropods are exposed to pesticides, arthropods may develop, ultimately, hormesis and/or resistance responses to such chemicals (Kendig et al., 2010; Liang et al., 2012). In our experiment assessing the transgenerational effects in the F1 generation of *B. brassicae*, we showed that the exposure to the LC_5_ and LC_15_ of spirotetramat in the parent generation (F0) significantly affected the F1 generation population growth, notably through an increased duration of the preadult stage, of TPOP, and of the mean generation time (*T*). These effects translate to a lower intrinsic rate of increase (*r*_*i*_), finite rate of increase (*λ*), net reproductive rate (*R*_0_). Such effects on population growth have been reported when treated with lethal and sublethal concentrations of various insecticides such as also in *A. gossypii* (Chen et al., 2016), *M. persicae* (Tang et al., 2015) *H. variegata* (Goeze) (Rahmani and Bandani, 2013) and *Plutella xylostella* (Guo et al., 2013). In general, the life table parameters estimated in this study are somewhat similar to the published data on different insect pests including aphids (Chen et al., 2016; Ullah et al., 2019; Hafeez et al., 2021a). The results of the current study suggest that sublethal concentrations of spirotetramat reduced the productiveness of the parent generation (F_0_) of *B. brassicae* and had a transgenerational effect on the descendants by extending the preadult developmental length, reducing the survival rate of undeveloped phases and also overwhelming the fecundity of F_1_ generation.

## Conclusion

Sublethal concentrations can interfere with the growth and overwhelm the population growth of the *B. brassicae* offspring. In practice, the results of the present study (under laboratory conditions) stressed the importance of assessing sublethal effects of the pesticide on this *B. brassicae* and also assessing how these effects may translate to the population level in the field. Therefore, further studies using various low-lethal and sublethal concentrations may be needed to provide a more comprehensive evaluation of putative hormesis responses to spirotetramat in *B. brassicae*. Our study hinted at the need to study further possible effects of spirotetramat on *B. brassicae,* in the aim to develop optimized IPM packages including this new pesticide (Table 4).

## Data Availability

The original contributions presented in the study are included in the article/supplementary material, further inquiries can be directed to the corresponding authors.
