# Erratum: Assessment of sublethal and transgenerational effects of spirotetramat, on population growth of cabbage aphid, *Brevicoryne brassicae*L. (Hemiptera: Aphididae)

**DOI:** 10.3389/fphys.2023.1192013

**Published:** 2023-03-31

**Authors:** Frontiers Production Office

**Affiliations:** Frontiers Media SA, Lausanne, Switzerland

**Keywords:** cabbage aphid, population growth, spirotetramat, sublethal concentrations, transgenerational effects

Due to a production error, one of the **Author’s names** was spelt incorrectly. The incorrect spelling is “Yassor Sabry Mostafa”. The correct spelling is “Yasser Sabry Mostafa”.

The publisher apologizes for the mistake.

The original article has been updated.

